# Novel Lignan and Stilbenoid Mixture Shows Anticarcinogenic Efficacy in Preclinical PC-3M-luc2 Prostate Cancer Model

**DOI:** 10.1371/journal.pone.0093764

**Published:** 2014-04-03

**Authors:** Emrah Yatkin, Lauri Polari, Teemu D. Laajala, Annika Smeds, Christer Eckerman, Bjarne Holmbom, Niina M. Saarinen, Tero Aittokallio, Sari I. Mäkelä

**Affiliations:** 1 Functional Foods Forum, University of Turku, Turku, Finland; 2 Turku Center for Disease Modeling (TCDM), Institute of Biomedicine, University of Turku, Turku, Finland; 3 Department of Mathematics and Statistics, University of Turku, Turku, Finland; 4 Åbo Akademi University, Process Chemistry Centre, Laboratory of Wood and Paper Chemistry, Turku, Finland; 5 Institute for Molecular Medicine Finland (FIMM), University of Helsinki, Helsinki, Finland; University of Colorado Denver, United States of America

## Abstract

Prostate cancer is the most common cancer of men in the Western world, and novel approaches for prostate cancer risk reduction are needed. Plant-derived phenolic compounds attenuate prostate cancer growth in preclinical models by several mechanisms, which is in line with epidemiological findings suggesting that consumption of plant-based diets is associated with low risk of prostate cancer. The objective of this study was to assess the effects of a novel lignan-stilbenoid mixture in PC-3M-luc2 human prostate cancer cells *in vitro* and in orthotopic xenografts. Lignan and stilbenoid –rich extract was obtained from Scots pine (*Pinus sylvestris*) knots. Pine knot extract as well as stilbenoids (methyl pinosylvin and pinosylvin), and lignans (matairesinol and nortrachelogenin) present in pine knot extract showed antiproliferative and proapoptotic efficacy at ≥40 μM concentration *in vitro*. Furthermore, pine knot extract derived stilbenoids enhanced tumor necrosis factor-related apoptosis-inducing ligand (TRAIL) induced apoptosis already at ≥10 μM concentrations. In orthotopic PC-3M-luc2 xenograft bearing immunocompromized mice, three-week peroral exposure to pine knot extract (52 mg of lignans and stilbenoids per kg of body weight) was well tolerated and showed anti-tumorigenic efficacy, demonstrated by multivariate analysis combining essential markers of tumor growth (i.e. tumor volume, vascularization, and cell proliferation). Methyl pinosylvin, pinosylvin, matairesinol, nortrachelogenin, as well as resveratrol, a metabolite of pinosylvin, were detected in serum at total concentration of 7−73 μM, confirming the bioavailability of pine knot extract derived lignans and stilbenoids. In summary, our data indicates that pine knot extract is a novel and cost-effective source of resveratrol, methyl pinosylvin and other bioactive lignans and stilbenoids. Pine knot extract shows anticarcinogenic efficacy in preclinical prostate cancer model, and our *in vitro* data suggests that compounds derived from the extract may have potential as novel chemosensitizers to TRAIL. These findings promote further research on health-related applications of wood biochemicals.

## Introduction

Prostate cancer (PCa) is the most common cancer of men in North America, Western Europe, Eastern Europe, and Scandinavia. The long natural history of PCa presents a relatively wide time window for dietary or pharmacological interventions that could manifest as reduction in incidence, recurrence, morbidity or progression of the disease [1]. Proposed modifiable risk factors for PCa that provide potential targets for prevention include inflammation, steroid hormones and their receptors, obesity, hypercholesterolemia and dietary factors [2]. Natural phenolic compounds, possessing anticarcinogenic properties, may offer interesting possibilities for the reduction of cancer burden [3], [4]. However, more data on their efficacy and mechanisms of action is needed from relevant preclinical models, in order to select the optimal compounds or mixtures for clinical trials and product development.

Trees are an abundant source of phenolic compounds, structurally identical or similar to those in edible plants [5]. These compounds can easily be isolated from wood by hydrophilic extraction after removing the lipophilic extractives by hexane extraction [6]. Wood-derived extracts are thus a cost-effective source of natural phenolic compounds, and an attractive option for the development of novel health-promoting products.

Plant phenolics, such as lignans and stilbenoids, modulate several important biological processes in mammalian cells [7], [8], and show anticarcinogenic properties in preclinical PCa models. Rye or flaxseed-containing diets, which have a high lignan content, as well as a diet supplemented with a wood-derived plant lignan, 7-hydroxymataresinol (HMR), inhibit the growth of PCa in *in vivo* [9], [10], [11], [12]. Similarly, plant-derived stilbenoids, resveratrol (RV) and pterostilbene have been reported to suppress the growth of PCa *in vivo* [13]. Several possible mechanisms, by which lignans and stilbenoids may exert their anticarcinogenic actions in PCa have been identified, including cell cycle arrest and induction of apoptosis [14], [15], inhibition of cell proliferation [16], [17], tumor vascularization [18], modulation of cytokine profile [19], and sensitization of cancer cells to programmed cell death [20], [21], [22].

Tumor Necrosis Factor-Related Apoptosis-Inducing Ligand (TRAIL) –mediated apoptosis has been recently identified as an interesting target for lignans and stilbenoids. TRAIL is a protein functioning as a ligand, which activates a death-signaling pathway especially in cancer cells, with minimal toxicity to normal tissues [23]. Resistance to TRAIL is common in PCa, and compounds that sensitize PCa to TRAIL are currently under active investigation. Interestingly, lignans, such as matairesinol (MR), and nortrachelogenin (NTG), as well as RV enhance the antitumor activity of TRAIL in PCa cells [21], [22] or xenografts [18].

Experimental studies thus suggest that natural lignans and stilbenoids attenuate PCa growth via numerous mechanisms of action. It is, therefore, plausible to propose that combination of the two groups of phenolic compounds may offer additional benefits over the use of single compounds. However, no published data exists on the combined effects of lignans and stilbenoids in controlled experimental settings. In this study, we demonstrated growth-inhibitory effects of pine knotwood extract (PKE), rich in lignans and stilbenoids, in PC-3M-luc2 human PCa cells *in vitro* and *in vivo*. Furthermore, we confirmed the bioavailability of PKE-derived phenolics, and identified the compounds likely to account for the anticarcinogenic effects of PKE.

## Materials and Methods

### Ethics Statement

Animal care and use was conducted in accordance with the Finnish Act on Animal Experimentation and EU laws, guidelines, and recommendations. All studies were approved by the national Animal Experiment Board in Finland (License number 1993/04.10.03/2011).

### Preparation and Chemical Characterization of PKE

Scots pine knots were obtained from an industrial pulp mill process (so-called stone trap) (UPM Tervasaari, Valkeakoski, Finland). The knots were ground, screened (<2 mm), freeze-dried, and extracted sequentially with *n*-hexane at 90°C (3×5 min) and ethanol-water (95∶5 by vol.) at 100°C (3×5 min) using accelerated solvent extraction. The PKE was chemically characterised by GC-flame ionization detection (FID), GC-MS and by high-performance size-exclusion chromatography with evaporative light-scattering detection (HPSEC-ELSD). The GC-FID, GC-MS, and HPSEC-ELSD analyses were performed as described previously [24]. GC-FID was used for quantification of the components in the extract that eluted from the GC column. The quantification was based on the triplicate analyses of the same extract. Heneicosanoic acid was used as internal standard, with the response factors 1.00. The individual compounds detected by GC-FID were identified by GC-MS, using commercial and the laboratory’s own spectral libraries. The molar mass distribution of the PKE components was determined by HPSEC-ELSD.

The major compound groups in the PKE were: lignans (16%), stilbenoids (17%), oxidized resin acids (20%), resin acids (24%), and higher-molar-mass compounds (550–4000 Da, 18%). The concentrations (wt-% of dry extract) of the main GC-detectable phenolics in the PKE were pinosylvin monomethyl ether (MePS, 10.2%), NTG (7.0%), pinosylvin (PS, 4.0%), MR (1.5%), abietic acid (1.5%), and pinostilbene (0.4%). The GC-detectable resin acids accounted for 21.8% of the dry weight of the PKE. Minor amounts (<0.1–0.5%) of lignans HMR, conidendric acid, secoisolariciresinol and todolactol A were found.

### Reference and Test Compounds

The preparation and purities of the lignans HMR, MR, NTG, secoisolarici-, larici-, cyclolarici-, 7-oxomatai-, pino-, and medioresinol, α-conidendrin, enterolactone, 7-hydroxyenterolactone, MR-*d*_6_, and enterolactone-*d*_6_ have been described previously [25], [26]. The stilbenes PS and MePS and the resin acid dehydroabietic acid (purities >95%) were prepared in the Laboratory of Wood and Paper Chemistry at Åbo Akademi University by isolation from wood and purification by flash chromatography (Biotage Flash 40i) with silica columns. RV and enterodiol were from Sigma-Aldrich Co, pinostilbene from TCl Pharma Chem., Inc (Vaughan, ON, Canada) and *O*-methylpodocarpic acid, neoabietic acid and abietic acid from Helix Biotech Corp. (CanSyn Chem Corp., Toronto, Canada).

In order to study the combined effects of pure PKE-derived lignans and stilbenoids *in vitro*, we prepared a lignan-stilbene mixture (LS mixture) containing the four most abundant PKE-derived compounds in molar ratios similar to PKE (Table 1).

**Table 1:** Concentrations of four main lignans and stilbenoids in pine knot extract (PKE) and composition of lignan-stilbenoid (LS) mixture.

Polyphenol	PKE	PKE (40 mg/l)	LS mixture (40 μM)
	(w/w %)	(μM)	(μM)
**Methylpinosylvin**	10	20	21.4
**Nortrachelogenin**	7	7	6.8
**Pinosylvin**	4	10	9.6
**Matairesinol**	1.5	2	2.2

### Cell Culture and Proliferation, Apoptosis and Cell Cycle Assays

PC-3M-luc2 cells (Caliper Life Sciences, Hopkinton, MA) were cultured in Dulbeccos’s phenol red-free modified Eagle medium supplemented with 50 IU/ml penicillin, 50 μg/ml streptomycin, 2 mM L-glutamine, and 10% heat-inactivated fetal bovine serum (Invitrogen, Paisley, UK). The cells were maintained in a humidified 5% CO_2_/air atmosphere at 37°C.

For cell proliferation assay, PC-3M-luc2 cells were seeded into a 96-well plate (1500 cells per well). Dimethyl sulfoxide stock solutions of PKE (1–100 mg/l), purified compounds (1–100 μM) or dimethyl sulfoxide controls prepared in culture medium were added to wells (six replicates). After 48 hours, cell proliferation was measured with Cell Proliferation ELISA BrdU immunoassay kit (Roche Diagnostics, Mannheim, Germany) according to the manufacturer’s instructions.

For apoptosis assay, PC-3M-luc2 cells were seeded into a 96-well plate. When 60–80% confluent half of the medium was removed and replaced with the medium containing the wood-derived compounds (final concentrations 1–40 mg/l). After 48 hours, cells were detached with trypsin and disrupted with 0.4 M citrate buffer in PBS containing 0.3% triton-X (Sigma Life Sciences, St. Louis, MO). Nuclei and nuclear fragments were labeled with propidium iodide (Sigma). To determine the fraction of sub-G0/G1 events, samples were analyzed with flow cytometer (BD LSR II, BD Biosciences, Franklin Lakes, NJ, USA). For assessment of TRAIL sensitivity, cells were treated with combination of wood derived compounds (as above) and human TRAIL/Apo2L (cat. no. C-63600, Promokine, Heidelberg, Germany) at concentrations of 3, 25, 50, and 100 ng/ml.

### Orthotopic PC-3M-luc2 Xenografts in Athymic Mice

Hsd:Athymic Nude – Foxn1^nu^ male mice (5–6-week-old) purchased from Harlan Laboratories (Horst, The Netherlands) were housed in aseptic conditions in the Central Animal Laboratory of the University of Turku. The mice were acclimatized for two weeks; provided with soy-free natural ingredient diet (RM3, SDS, Essex, UK) and tap water *ad libitum*.

PC-3M-luc2 cells used in orthotopic xenograft experiment were cultured in RPMI 1640 phenol red-free medium supplemented with 50 IU/ml penicillin, 50 μg/ml streptomycin, 2 mM L-glutamine, and 10% heat inactivated fetal bovine serum. The cells were maintained in a humidified 5% CO_2_/air atmosphere at 37°C. PC-3M-luc2 cells (1×10^6^ cells in 20 μl plain RPMI 1640 medium) were inoculated into the dorsolateral prostate through an abdominal incision under isoflurane anesthesia. For pain relief, mice were given buprenorphine (Temgesic, 0.05–0.1 mg/kg *s.c*.) and carpofen (Rimadyl, 5 mg/kg *s.c*.) before and after the operation, respectively. Tumor growth was followed by bioluminescence imaging with IVIS lumina II, equipped with XGI-8 Gas Anesthesia System (Caliper Life Sciences, Runcorn, UK). Mice were anesthetized with isoflurane, injected *i.p.* with 150 mg/kg D-luciferin (Xenogen cat. no. XR-1001, Oregon, USA) 10 min prior to imaging, and bioluminescence was quantified using LivingImage 4.09A Carbon software (Xenogen).

One week after inoculation, tumor-free animals were identified by bioluminescence imaging and excluded from the study. Tumor bearing mice were allocated into vehicle (control), low dose (32 mg/kg) or high dose (160 mg/kg) PKE groups. Low and high dose contained approximately 5.0 and 5.4, and 25 and 27 mg of lignans and stilbenoids, respectively. PKE was dissolved in a vehicle containing 10% (v/v) of absolute ethanol and 90% (v/v) corn oil. Both vehicle and PKE were gavaged *p.o.* daily. Mice were weighed weekly, and bioluminescence imaging was carried out twice a week.

During the third week of treatment 24-h urine samples were collected in metabolic cages (5–6 mice per cage). Urine was collected in jars containing 0.15 M sodium azide and 0.56 M ascorbic acid as preservatives. Urine samples were centrifuged and stored in −20°C until analyzed.

After three-week treatment, the mice were sacrificed with CO_2_ suffocation followed by cervical dislocation. Blood was collected via heart puncture, centrifuged, and the serum was separated and kept in −70°C until analyzed. The tumors were dissected and weighed, and the tumor size was measured with a caliper in three dimensions. The tumor volume was calculated using the formula: volume (mm^3^) = (length × width × height) × π/6. The tumors were fixed in 10% neutral buffered formalin and processed for paraffin embedding and used for immunohistochemical analyses.

### Analysis of Lignans and Stilbenoids Mouse Serum and Urine

Serum (50 μl) and urine (300 μl) samples were hydrolyzed and solid-phase extracted as previously described with some modifications [25], [27]. For hydrolysis, a freshly prepared β-glucuronidase/−sulphatase in 10 mM of sodium acetate buffer (pH 5.0) was added to serum and urine samples and incubated for 19 h at 37°C. After the hydrolysis, internal standards were added to the samples: MR-*d*_6_ for plant lignans and stilbenes, EL-*d*_6_ for enterolignans (final concentrations ∼1 μg/ml), and *O*-methylpodocarpic acid for the resin acids (final concentration ∼3 μg/ml). The solid-phase extracted and evaporated serum and urine samples [25] were redissolved in 150 μl and 500 μl methanol/0.1% acetic acid 20∶80 (v/v), respectively. The PKE components and their metabolites were quantified using HPLC-MS/MS with multiple ion monitoring in the negative ion mode as described previously [24]. The optimized multiple ion monitoring transitions were the deprotonated molecular ion and the following fragments in MS^2^ (*m/z*); PS 169 18; MePS 182 20; RV 143, 22; pinostilbene 225. The resin acids formed no fragments in MS^2^. The used cone voltages were 40 V for the stilbenes and 48 V for the resin acids. The collision energies were around 20 eV for the stilbenes and 5.0 eV for the resin acids. The MS parameters for the analyzed lignans have been described previously [26]. The quantifications were carried out using standard solutions containing the internal standards and six concentration levels of the analytes as described previously [26].

### Immunohistochemistry and Detection of Apoptotic Cells in Tumor Samples

For von Willebrand factor (vWF, dilution: 1∶4000, ab6994-100, Abcam, Cambridge, U.K.) and phospho-Histone H3 (pH-H3, dilution: 1∶200, Ser10, Cell signaling) immunohistochemistry, tissue section were deparaffinized, rehydrated, and incubated in 10 mM Tris-EDTA pH 9 (for vWF staining) or in 10 mM sodium citrate pH 6 (for pH-H3 staining) buffer, in a microwave oven for antigen retrieval. The endogenous peroxidase activity was blocked with 3% H_2_O_2_. After rinsing with PBS, the sections were incubated with 3% bovine serum albumin (BSA) for 10 minutes, and labeled with vWF or pH-H3 antibodies for 60 minutes at room temperature (RT) or overnight at +4°C, respectively. Horseradish peroxidase labeled polymer anti-rabbit (DAKO envision system, Dako) was used as a secondary antibody. Diaminobenzidine (Dako) was applied to the sections followed by Mayer’s hematoxylin and mounting.

The terminal deoxynucleotidyl transferase biotin-dUTP nick end labeling (TUNEL) method was used to determine apoptotic cells in tumor sections. The analysis was performed using ApopTag Peroxidase in situ Apoptosis Detection Kit (Chemicon International, Hampshire, UK) according to the manufacturer’s instructions. The sections were deparaffinized, rehydrated and treated with 10 mM sodium citrate buffer in microwave for antigen retrieval. The endogenous peroxidase activity was blocked with 3% H_2_O_2_. For positive control, one section was incubated with DNase I (Invitrogen) for 30 min at +37°C. For the TUNEL, sections were incubated with 0.8 U/μl TdT reaction mix: TdT buffer, 4 U/μl transferase, 25 mM CoCl_2_ (Terminal transferase, Roche, Mannheim, Germany), biotin-16-dUTP (Roche) and MilliQ water; and for the negative control, one section was incubated with TUNEL reaction mix without transferase for one hour at +37°C. TUNEL reaction was stopped with 300 mM NaCl, 30 mM sodium citrate in dH_2_O (15 min at RT). Nonspecific sites were blocked with 3% BSA/PBS for 30 min at RT. ExtrAvidine®-Peroxidase 1∶500 (Sigma) was applied in 1% BSA/PBS for 30 min at +37°C, after which diaminobenzidine was applied and the sections were stained with Mayer’s hematoxylin and mounted.

### Quantification of Proliferation and Apoptosis Indices, and Blood Vessel Density and Length

Stained sections were scanned with a virtual microscope (Digital Virtual Microscope, Soft Imaging System, Olympus, Germany). The number and length of vWF-positive vessels was quantified in three or four selected areas by using the measurement tool of dotSlide program (Soft Imaging System). The quantification of pH-H3 and TUNEL-positive cell in tumor sections was carried out with custom functions developed for tissue image analysis by Quva Ltd (Tampere, Finland). First, the images obtained from scanned slides were normalized for intensity and color variations. Processing was continued by image thresholding, which detects areas with suitable color to be considered as the positively stained target cells. Finally, extraneous detections were filtered out by size and shape information, and the resulting cells were visualized in red spots over the original image.

### Statistical Analyses

The univariate analyses were performed using GraphPad Prism version 4.00 for Windows (GraphPad Software Inc., San Diego, CA). In normally distributed data, the one-way analysis of variance and Tukey’s post-hoc test were used; otherwise, the Kruskal-Wallis or non-parametric t-test was used. All data are presented as group mean±SEM. Differences were considered statistically significant at *p*<0.05.

Pairwise multivariate comparisons of group means (say µ1 and µ2) were tested with the Mahalanobis distance (MD):
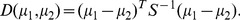
(1)


Mahalanobis distance is a well-known multivariate statistic, which incorporates linear correlations of covariates through the covariance-variance matrix *S*, with dimension equal to the number of covariates [28]. By accumulating complementary information from interconnected factors and having sensitivity to patterns differing from the major trends [29], MD is a convenient statistic for testing more subtle treatment effects potentially missed by the univariate tests. In special cases, MD reduces to the standardized Euclidean distance, if *S* is a diagonal matrix, and to the ordinary Euclidean distance, if *S* is a unit matrix, and thus links closely to the conventional *t* statistic testing of independent tumor growth markers. Statistical significance of the distance *D* was evaluated by random permutation of the class labels in the two-sample tests (Vehicle vs. Low dose or Vehicle vs. High dose) [30]. The null distributions were obtained by running 100,000 simulations of such randomly generated distances and the empirical *p*-values were defined as the proportion of random distances greater than or equal to the observed distance *D*.

## Results

### PKE and its Components Inhibit PC-3M-luc2 Cell Proliferation and Induce Apoptosis *in vitro*

**Figure 1:**
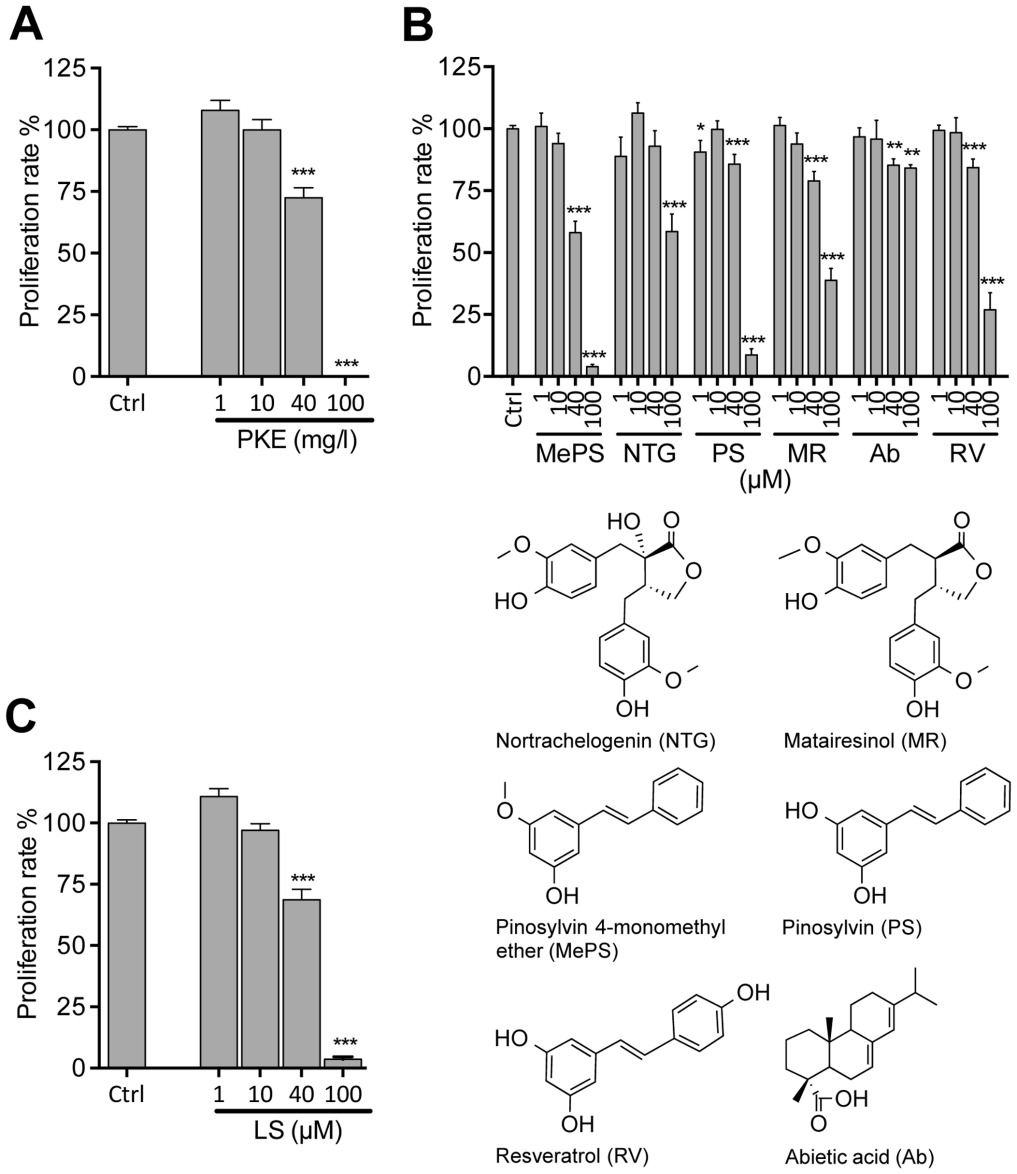
Pine knot extract (PKE) and its polyphenols inhibit the proliferation of PC-3M-luc2 prostate cancer cells. A) Cells were treated with PKE or a mixture of its main lignans and stilbenoids (LS mixture) for 48 hours. B) Cells were treated with individual PKE derived compounds for 48 hours. Cell proliferation rate expressed as percent of control was measured with BrdU assay. The data are means of three independent experiments ± SEM. Statistical significance was determined by non-parametric t-test for each treatment in comparison to respective vehicle treatment (Ctrl). *p<0.05, **p<0.01, ***p<0.001.

**Figure 2:**
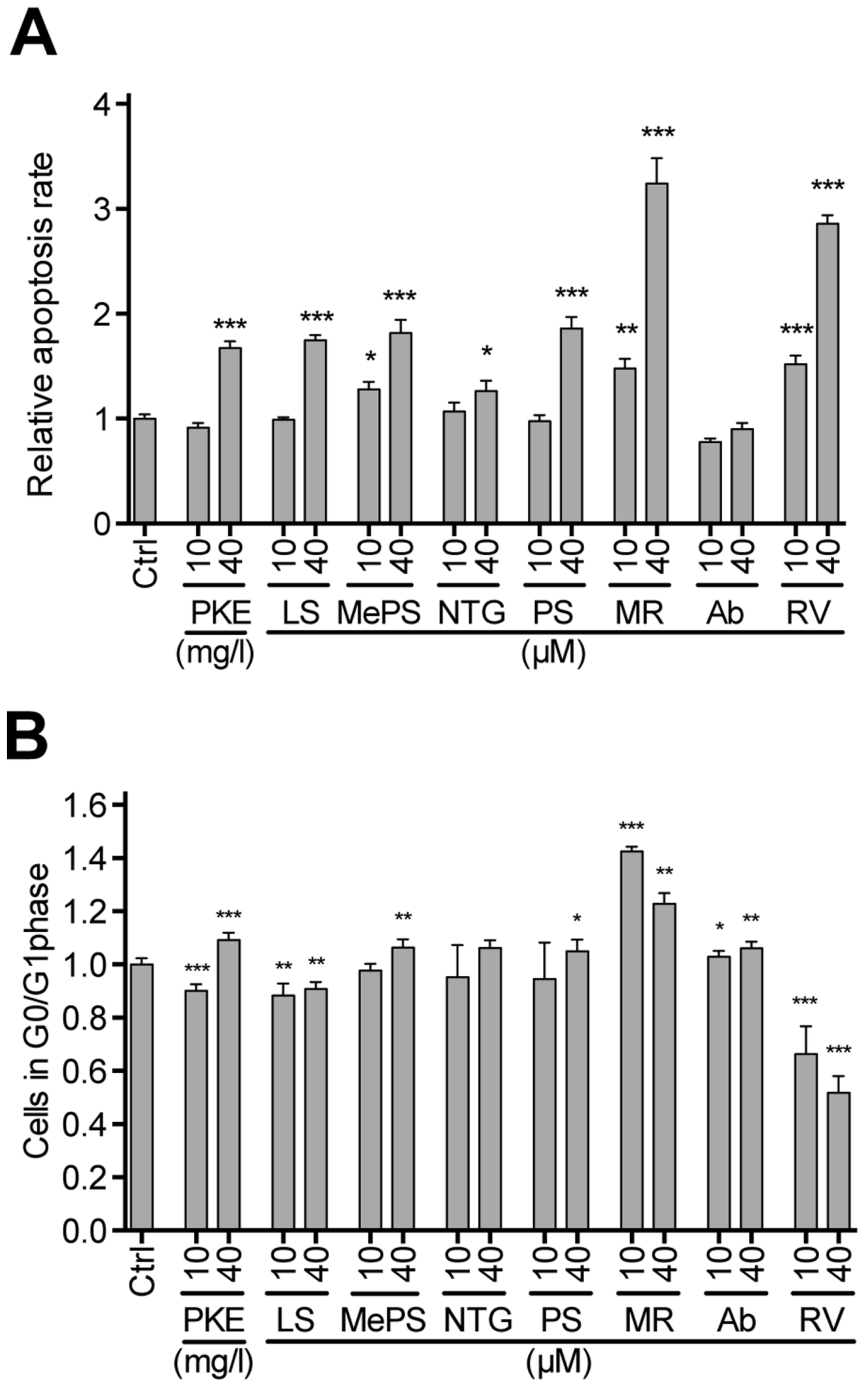
PKE and its compounds induce A) apoptosis and B) G0/G1 cell cycle arrest of PC-3M-luc2 cells *in vitro.* Apoptosis rates and number of cells in G0/G1 phase are expressed relative to vehicle control (Ctrl = 1). The cells were treated with test compounds for 48 hours and apoptosis rate and cell cycle phase was determined with flow cytometer. LS, mixture of main PKE lignans and stilbenes; MePS, pinosylvin monomethyl ether; NTG, nortrachelogenin; PS, pinosylvin; MR, matairesinol; Ab, abietic acid; RV, resveratrol. The data are means of three independent experiments ± SEM. Statistical significance was determined by non-parametric t-test for each treatment in comparison to respective Ctrl. *p<0.05, **p<0.01, ***p<0.001.

**Figure 3:**
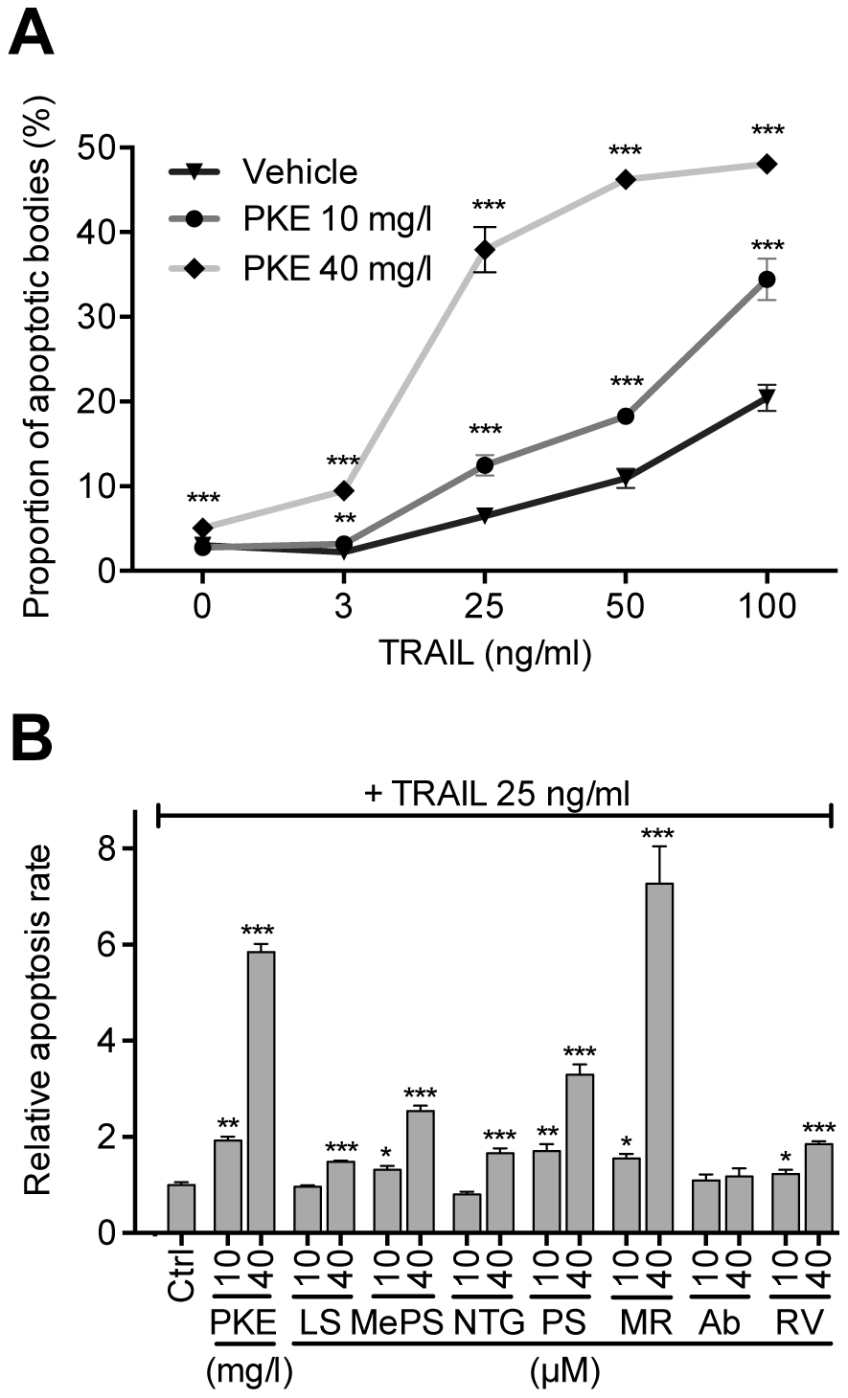
PKE and its compounds sensitize PC-3M-luc2 cells to TRAIL -induced apoptosis *in vitro.* A) Cells treated with PKE in combination with increasing concentrations of human TRAIL (0−100 ng/ml), values are percentages of nuclear fragmentation. B) Cells treated with PKE, LS mixture, or with individual PKE compounds in combination with human TRAIL (25 ng/ml), values are relative apoptosis rates (Ctrl = 1). The cells were treated with test compounds for 48 hours and nuclear fragmentation was determined with flow cytometer. LS, mixture of main PKE lignans and stilbenes; MePS, pinosylvin monomethyl ether; NTG, nortrachelogenin; PS, pinosylvin; MR, matairesinol; Ab, abietic acid; RV, resveratrol. The data are means of three independent experiments ± SEM. Statistical significance was determined by non-parametric t-test for each treatment in comparison to respective vehicle treatment (Ctrl). *p<0.05, **p<0.01, ***p<0.001.

PKE significantly attenuated PC-3M-luc2 cell proliferation at ≥40 mg/l (Fig. 1A), demonstrated by reduced incorporation of BrdU. In line with this, the main constituents of PKE, stilbenoids (MePS and PS), as well as lignans (MR and NTG) each significantly inhibited cell proliferation at 40–100 μM concentration (Fig. 1B). Furthermore, combination of these four compounds (MePS, PS, MR, and NTG), in the same ratio as present in PKE (lignan-stilbene, LS, mixture), reduced BrdU incorporation in a manner similar to the original extract (Fig. 1C). PKE (40 mg/l) and LS mixture (40 μM), both increased apoptosis rate, demonstrated by flow cytometric analysis (Fig. 2A). PKE induced cell cycle arrest, i.e. increased the fraction of non-apoptotic cells in G0/G1 (Fig. 2B), and, respectively, decreased the fraction of cells in S and G2/M phases. Moreover, PKE (10 and 40 mg/ml) sensitized PC-3M-luc2 cells to TRAIL-induced apoptosis (Fig. 3A). LS mixture and all the tested PKE-derived compounds (MePS, NTG, PS, MR, and RV), except abietic acid, significantly enhanced TRAIL-mediated apoptosis (Fig. 3B), PS, MePS, and MR showing the most pronounced effects.

### Effect of PKE on Orthotopic PC-3M-luc2 Xenograft Volume, Cell Proliferation and Angiogenesis

**Figure 4:**
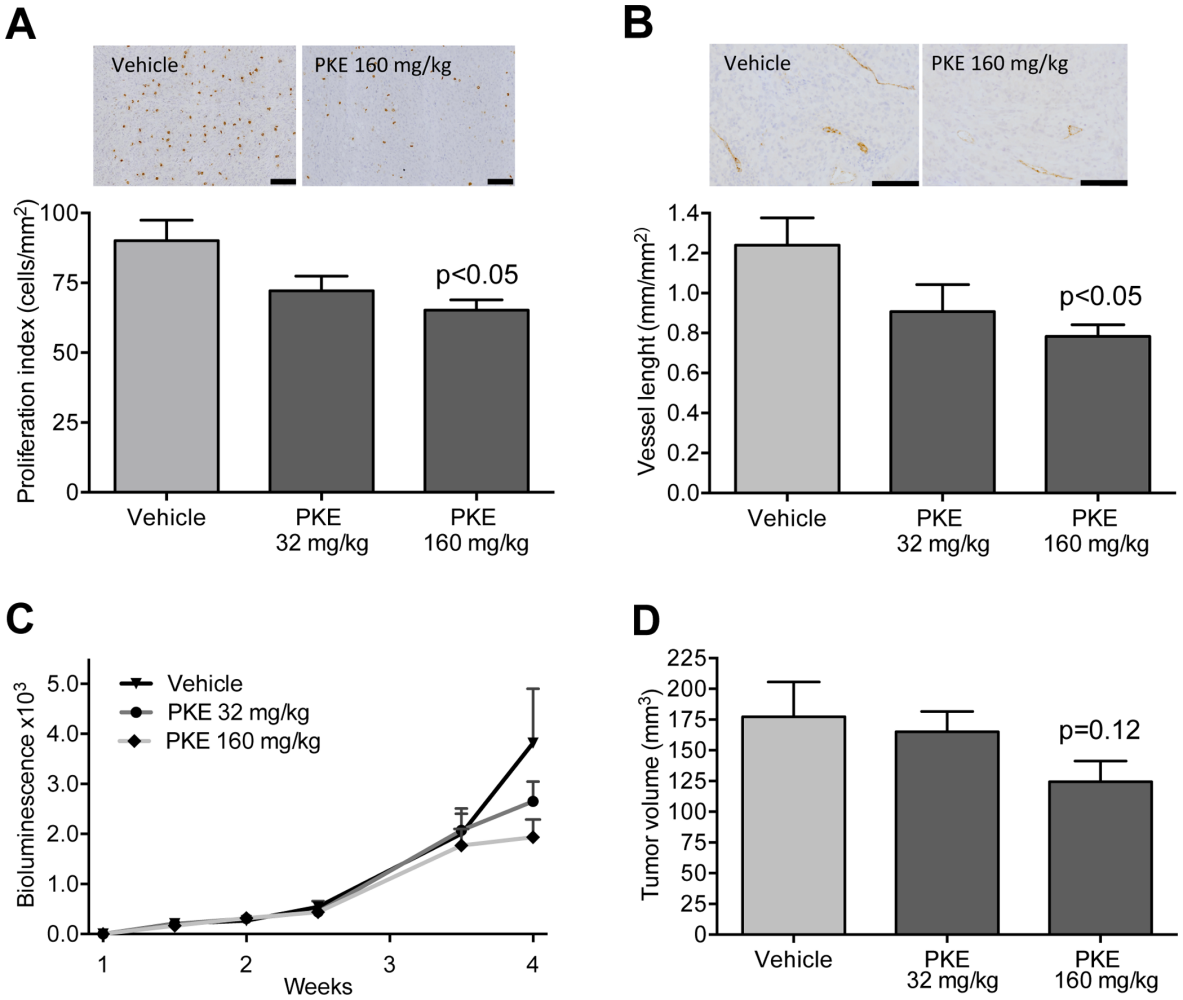
Peroral exposure of PKE attenuates growth of orthotopic PC-3M-luc2 xenografts. A) PKE decreases cell proliferation index. Representative images of pH-H3-immunostainings are shown. B) PKE decreases blood vessel length. Representative images of vWF-immunostainings are shown. C) Bioluminescence of the tumors, quantified biweekly by *in vivo* imaging. D) Tumor volumes at sacrifice. Mice were treated daily with vehicle (n = 11), PKE 32 mg/kg bwt (n = 10) or PKE 160 mg/kg bwt (n = 11) for three weeks. The data is expressed as mean ± SEM. Statistical significance was determined by one-way analysis of variance and Tukey’s post-hoc test. *p<0.05. Scale bars, 100 μm.

**Figure 5:**
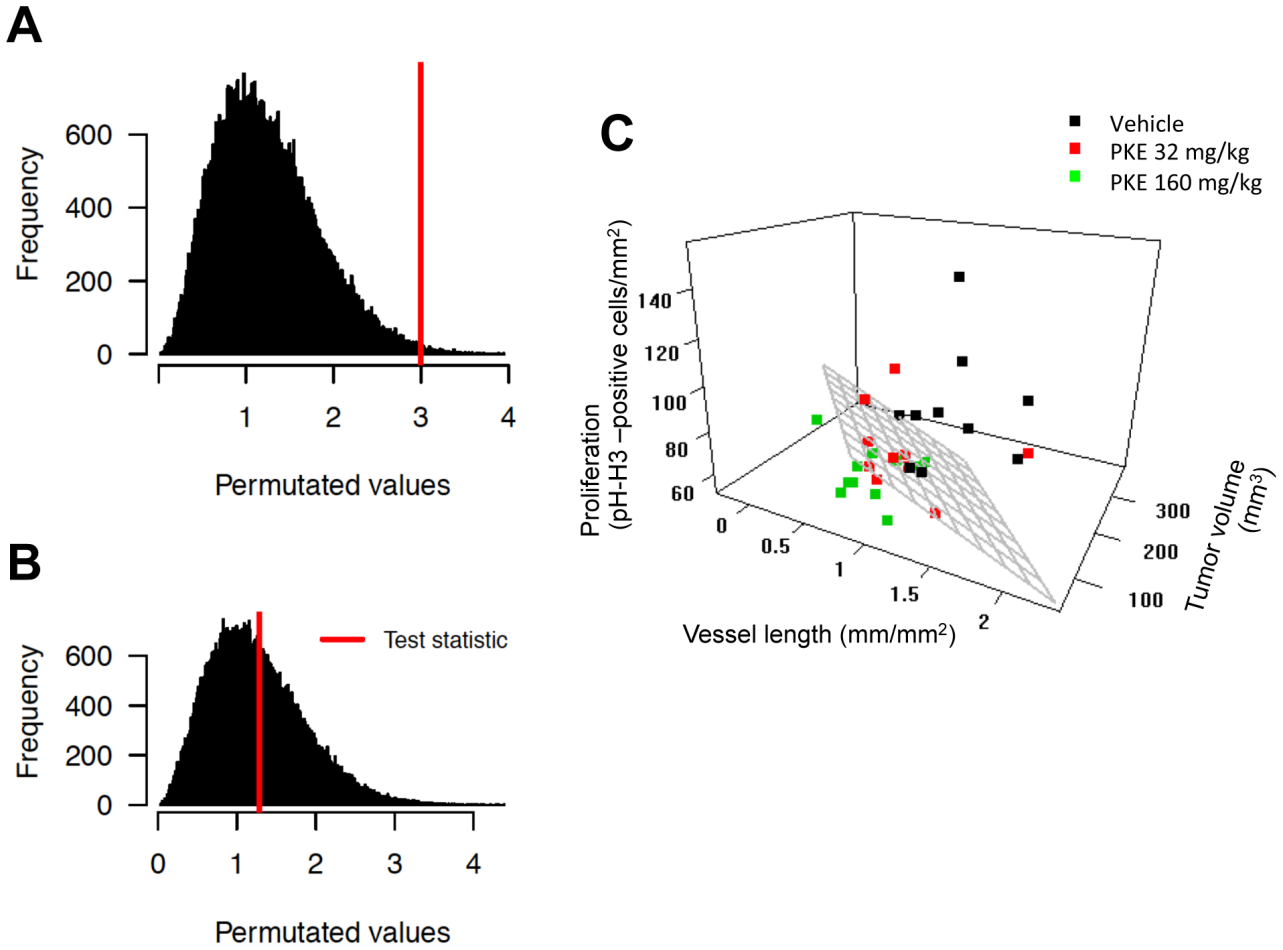
Multivariate testing and visualization of the relationships among vascularization, proliferation and tumor volume A) Vehicle vs. low dose comparison, and B) Vehicle vs. high dose comparison (p = 0.0051) using MD (p = 0.4195). C) Tumor volume, vessel length and proliferation index were detected as the most important factors of differences in the multivariate analyses. The hyperplane indicates that two of the groups (vehicle in black and high dose in green) were linearly separable when the three factors were considered together (Hyperplane: 100.55 = 0.104×Tumor volume +0.895×Proliferation index +21.1×Vessel length).

Three-week daily ingestion of PKE was well tolerated, and no signs of adverse effects were observed at any time point. PKE significantly reduced the proliferation index (Fig. 4A) and the length of blood vessels (i.e. perimeter) in the PC-3M-luc2 tumors (Fig. 4B). A trend to reduced bioluminescence and the PCa tumor volume was observed in the higher PKE dose (160 mg/kg) group (Fig. 4C and D). No significant changes were seen in the apoptosis index, as quantified from TUNEL-stained tumor sections (data not shown). The multivariate comparison of the excised tumor volume, log-transformed tumor bioluminescence, apoptosis and proliferation indices, and blood vessel density and length was performed using the MD statistic (Eq. 1). When the response was tested in terms of the combined effect of these 6 covariates (Fig. S1), there was a significant difference between the vehicle and high-dose PKE groups (p = 0.0051, Fig. 5A), while the vehicle and the low-dose PKE groups were similar (p = 0.4195, Fig. 5B). Interestingly, the combined evaluation of the covariates and their interactions clearly separated the vehicle and high-dose PKE groups (Fig. 5C, with the 3 most important features visualized). The inference drawn from this combined analysis was profoundly different from the independent univariate analyses (Fig. 4); for instance, the multivariate approach detected that some tumors had reduced vascularization (*vessel length*), while others had reduced size (*tumor volume*), and hence gave a multidimensional view of the treatment effects. The original tumor measurements are shown in the supplementary material as bivariate (Fig. S1) and univariate plots (Fig. S2). The calculated variance-covariance structure *S,* after scaling to correlation matrix, is shown in Fig. S3. As expected, the related factors, such as tumor volume and tumor bioluminescence or vessel density and vessel length, were highly correlated, and their distances were effectively grouped in the MD analysis (Fig. S3).

In order to evaluate those factors that were most essential for distinguishing the high-dose PKE group from the control group, an exhaustive testing of the MD for different combinations of the 6 covariates was performed. The most prominent effects were detected as a combination of tumor volume, proliferation index and vessel length. Investigation of the interactions among these three markers revealed that even if the individual covariates alone were not informative enough for distinguishing the two groups, when considered together, the vehicle and high-dose PKE groups were linearly separable with a hyperplane (Fig. 5C). Furthermore, albeit the vehicle and low-dose group difference was not statistically significant, the overall trend across the three groups suggested an increased antitumor effect with an increasing dose level (as seen in the order of the black, red and green clouds in Fig. 5C).

### Concentrations of Phenolic Compounds in the Serum and Urine of Tumor Bearing Mice

Micromolar concentrations of NTG, and MePS were detected in the serum of animals exposed to the higher dose of PKE (Table 2). Resin acids that were present in the PKE were not detected in the serum or urine samples. Interestingly, significant concentrations of RV, which is the monohydroxylated metabolite of PS, as well as pinostilbene, a metabolite of MePS, were detected in the serum and urine from mice fed with the PKE (Table 2). In addition, the concentrations of MR and HMR and their metabolites enterolactone, enterodiol, and 7-hydroxyenterolactone as well as concentrations of MePS, PS, NTG, and pinostilbene significantly increased compared to the control (Table 2). Stilbene and lignan metabolites were present in the serum 15 min−3 h after the last administration (data not shown), and the highest concentrations were measured at 2 h (Table 2). The metabolite profile in the urine (24 hour collection) was very similar to that in serum (data not shown).

**Table 2:** Concentrations of PKE-derived phenolic compounds in serum of PC-3M-luc2 orthotopic xenograft bearing mice.^a^

		Lignans and stilbenoids present in PKE					Metabolites of PKE-derived lignans and stilbenoids		
	n	NTG	MePS	PS	HMR	MR	PST	RV	EL
**Vehicle**	3	0.06±0.01	0.0±0.0	0.14±0.09	0.025±0.01	0.05±0.01	0.0±0.0	0.0±0.0	0.17±0.07
**PKE 160 mg/kg bwt**	6	3.2±1.1***	9.7±4.7***	2.2±0.3***	0.4±0.2***	0.8±0.3***	1.2±0.4**	5.8±4.0**	0.36±0.06*

a Tumor bearing male mice were gavaged with vehicle or PKE for 3 weeks. Terminal serum samples were collected 2–3 hours after the last gavage of PKE. NTG, nortachelogenin; MePS, pinosylvin monomethyl ether; PS, pinosylvin; HMR, 7-hydroxymatairesinol; MR, matairesinol; PST, pinostilbene; RV, resveratrol; EL, enterolactone. Concentrations (μM) are expressed as mean±SEM.

*p<0.05,

**p<0.01,

***p<0.001.

## Discussion

Epidemiological and preclinical studies suggest an inverse relationship between consumption of polyphenol-rich foods and the incidence of chronic diseases including cancer [3], [7], [31]. In particular, lignans and stilbenes, and their metabolites, are attractive candidates for PCa risk reduction. In this study, we have demonstrated anticarcinogenic effects of the Scots pine knot extract (PKE), a mixture rich in lignans and stilbenoids, *in vitro* and *in vivo* in an orthotopic PCa xenograft model.

Our results show that PKE, as well as its main lignan and stilbenoid components are antiproliferative and proapoptotic in PC-3M-luc2 cells *in vitro*. The effects of LS mixture, comprising of the main components of PKE (MePS, NTG, PS and MR) in molar ratios similar to PKE, are almost identical to those of PKE, indicating that these four phenolics are, indeed, the main bioactive components of PKE. Moreover, several of PKE-derived lignans and stilbenoids inhibited PC-3M-luc2 cell proliferation and induced apoptosis, when added alone. PKE and LS mixture and majority of individual PKE-derived phenolics showed significant effects at ≥40 μM, which is in the same range as has been reported for similar compounds before [20], [21], [32]. The total concentration range of bioactive PKE-derived phenolics (MePS, PS, NTG, MR, and the metabolite RV) in the serum of animals receiving the higher dose of PKE varied from 7 μM to 73 μM. This indicates that PKE-derived lignans and stilbenoids and their metabolites are bioavailable. Further, serum polyphenol concentrations were in the range found to reduce proliferation and increase apoptosis *in vitro* suggesting that the PKE-derived polyphenols may account for the anti-tumorigenic effect of PKE *in vivo*.

Two doses of PKE (32 and 160 mg/kg bwt) were tested in orthotopic PC-3M-luc2 xenograft model. It is known that xenograft studies display high variation in tumor growth, which sometimes makes it difficult to demonstrate the effects of dietary interventions [33]. The combined multivariate analysis that was employed in this study accounted for different patterns in the subtle treatment effects and the MD statistic was found to be more sensitive to detecting differences that were missed by the univariate statistical approaches. The non-aggressive nature of the PKE treatment, along with the interactions between the covariates and the underlying heterogeneity in the response patterns may be beyond the capability of the univariate analyses, but were effectively taken into the account here through the covariance structure *S* in MD and multidimensional comparisons of the group means. According to our analyses, the treatment effect became evident when interactions among multiple phenotypic readouts were combined, including tumor volume, vessel length and proliferation index, especially at a high enough dose of PKE. The lower dose corresponds roughly to a 1 g daily intake of plant phenolics, which, in humans, is achievable through a normal plant rich diet [34]. Significant effects on orthotopic PC-3M-luc2 xenograft growth were, however, seen only in the high-dose group, suggesting that supplementation of foods with plant phenolics may offer additional benefits as regards cancer risk reduction.

Furthermore, we demonstrated that PKE, LS mixture, and PKE-derived phenolics chemosensitize PC-3M-luc2 cells to TRAIL-mediated apoptosis *in vitro*. Resistance to TRAIL is common in cancers and considered a major obstacle for TRAIL-based clinical applications [35]. Sensitization to TRAIL-induced apoptosis have been earlier reported for several natural phenolic compounds, such as RV, fisetin, curcumin, MR, and NTG [21], [36], [37], [38], while this is the first study showing TRAIL-enhancing activity for pinosylvins *in vitro*. LS mixture and PKE-derived compounds, MePS, PS, MR and RV, were effective already at ≥10 μM concentrations *in vitro*. Total concentration of MePS, PS, MR and RV detected in the serum of PKE-fed animals was over 25 μM, suggesting that PKE, or compounds derived from PKE, may have potential as novel chemosensitizers to TRAIL. However, further studies with PKE or its compounds are warranted to demonstrate chemosensitization to TRAIL *in vivo*.

HPLC/GC analysis of urine and serum demonstrated that the main compounds in PKE, *i.e* MePS, PS, NTG, HMR, and MR, are bioavailable and excreted in urine, while resin acids, also present in PKE, were not detected. Interestingly, we found a number of novel as well as previously documented metabolites derived from PKE in the serum and urine in significant concentrations. These included pinostilbene (a novel identified metabolite of MePS), RV (a known metabolite of PS) [39], and enterolactone, 7-hydroxyenterolactone, and enterodiol (known metabolites of HMR and MR, respectively) [40]. PS, MePS, NTG and MR were present in the serum in high μM concentrations, i.e. in concentrations found to be effective *in vitro,* suggesting that these compounds may account for the observed anticancer effects also *in vivo*. Both lignans and stilbenoids are thus likely to contribute to the growth-inhibitory activity of PKE. Previous studies have demonstrated that lignans present in PKE (i.e. NTG and MR) or their metabolites (e.g. enterolactone) inhibit growth of PCa cells *in vitro* [22], [41], but it is not known if they are effective in PCa *in vivo*. Stilbenoids, such as RV and pterostilbene attenuate the growth of PCa xenografts [13], [42], [43], but there is no earlier published data on PS or its analogs in PCa models *in vitro* or *in vivo*. It was recently, however, reported that PS inhibits the growth of human colorectal cancer cells by suppressing Src/ERK and GSK-3/β-catenin signaling [32], and it is well possible that similar mechanisms may also be operative in PCa.

The serum and urine of PKE-fed mice contained RV, confirming earlier findings that PS is metabolized to RV *in vivo* [39]. This is of special interest regarding the development of PKE-based applications, as RV has been shown to inhibit PCa growth [42], [43], enhance antitumor activity of TRAIL in PCa *in vivo* [18], and to have favorable metabolic effects [8], [44]. There is a considerable interest in RV as a health-promoting agent, but development of RV-containing products and therapies has been hampered by its rapid metabolism, thus requiring very high or frequent dosing [4], [45]. PS, the likely precursor of RV in our study, constituted approximately 4% of the PKE, resulting in 6.4 mg/kg exposure of PS per day in high-PKE group. The fact that RV was not present in the PKE, but was found in the serum in micromolar concentrations hours after PKE gavage, demonstrates that oral administration of PKE is an attractive approach to reach bioactive concentrations of RV in serum. Furthermore, we demonstrated that MePS, which has not been studied before in PCa models, is well bioavailable from PKE, and exerts highly interesting biological effects at concentrations that can be reached *in vivo*.

In conclusion, we have demonstrated that orally administered PKE is well tolerated and inhibits the growth of orthotopic PCa xenografts. Based on our *in vitro* results, we suggest that PKE-derived lignans and stilbenoids and their metabolites account for the anticarcinogenic effect. We have identified a novel bioactive and bioavailable stilbenoid, MePS, and demonstrated that PKE is an abundant source on MePS, as well as other anticarcinogenic plant phenolics. Furthermore, serum RV concentration increases markedly after ingestion of PKE. Taken together, PKE is a novel and cost-effective source of RV, MePS and other potentially beneficial lignans and stilbenoids, promoting development of health-related applications of wood biochemicals.

## Supporting Information

Figure S1 Bivariate plots of the interactions between the tumor growth markers. Increasing the dose appears to shift the means of the groups to an expected direction. One measurement was missing for vessel density and vessel length in the vehicle group.
(TIF)

Figure S2 Univariate plots of the 6 end-point covariates. Means and medians are displayed in order to visualize the progressive trend for increasing dosage.
(TIF)

Figure S3 Covariance-variance matrix S scaled to a correlation matrix for visualization purposes. Agglomerative clustering of the factors illustrates that tumor volume and bioluminescence or vessel length and vessel density were correlated.
(TIF)
