# Supplementary figures and images for: Novel Lignan and Stilbenoid Mixture Shows Anticarcinogenic Efficacy in Preclinical PC-3M-luc2 Prostate Cancer Model
Source: PLoS One. 2014 Apr 3;9(4):e93764. doi: 10.1371/journal.pone.0093764 (PMC3974786; doi:10.1371/journal.pone.0093764)

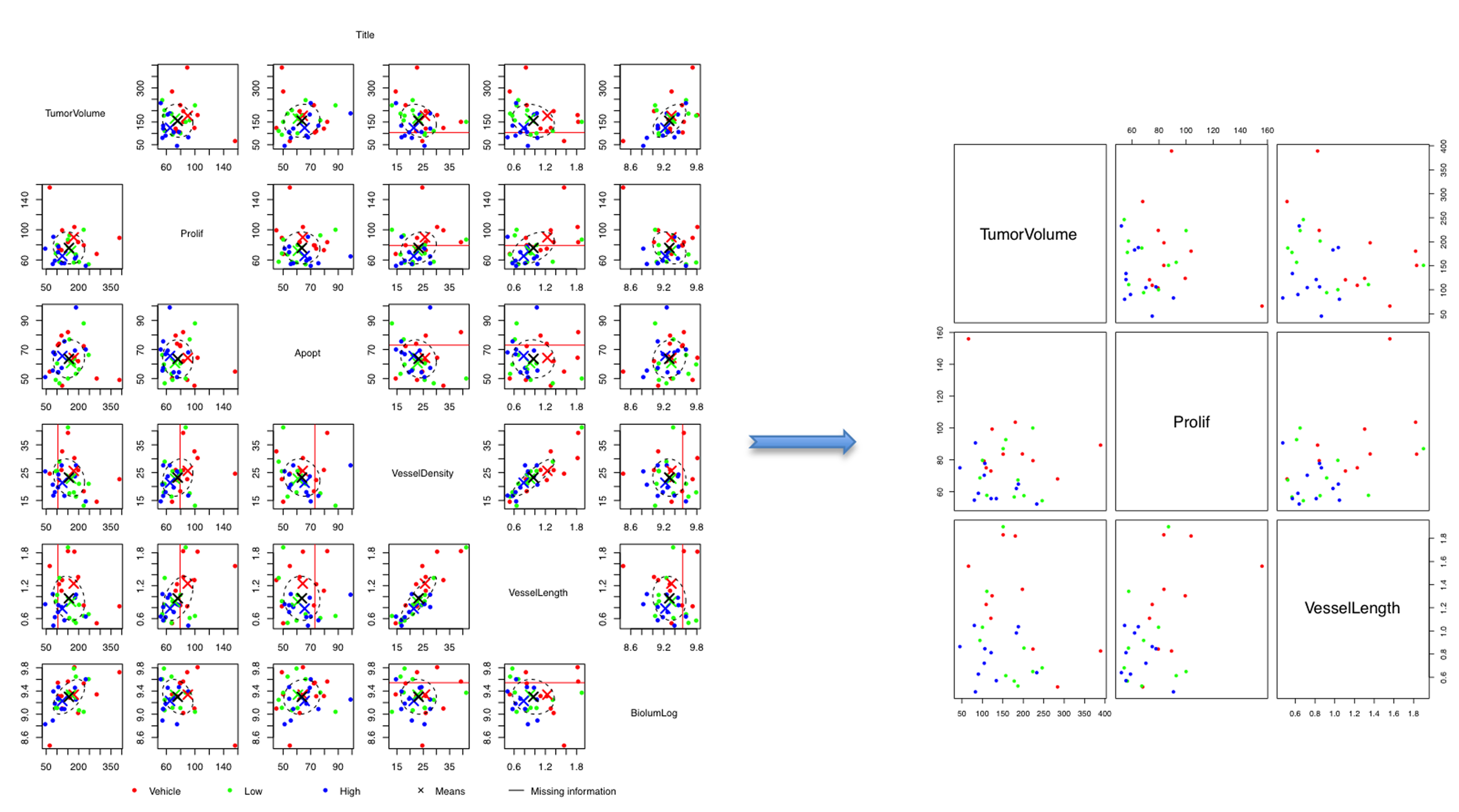

Supplement: Figure S1 — Bivariate plots of the interactions between the tumor growth markers. Increasing the dose appears to shift the means of the groups to an expected direction. One measurement was missing for vessel density and vessel length in the vehicle group. (TIF) [file pone.0093764.s001.tif]

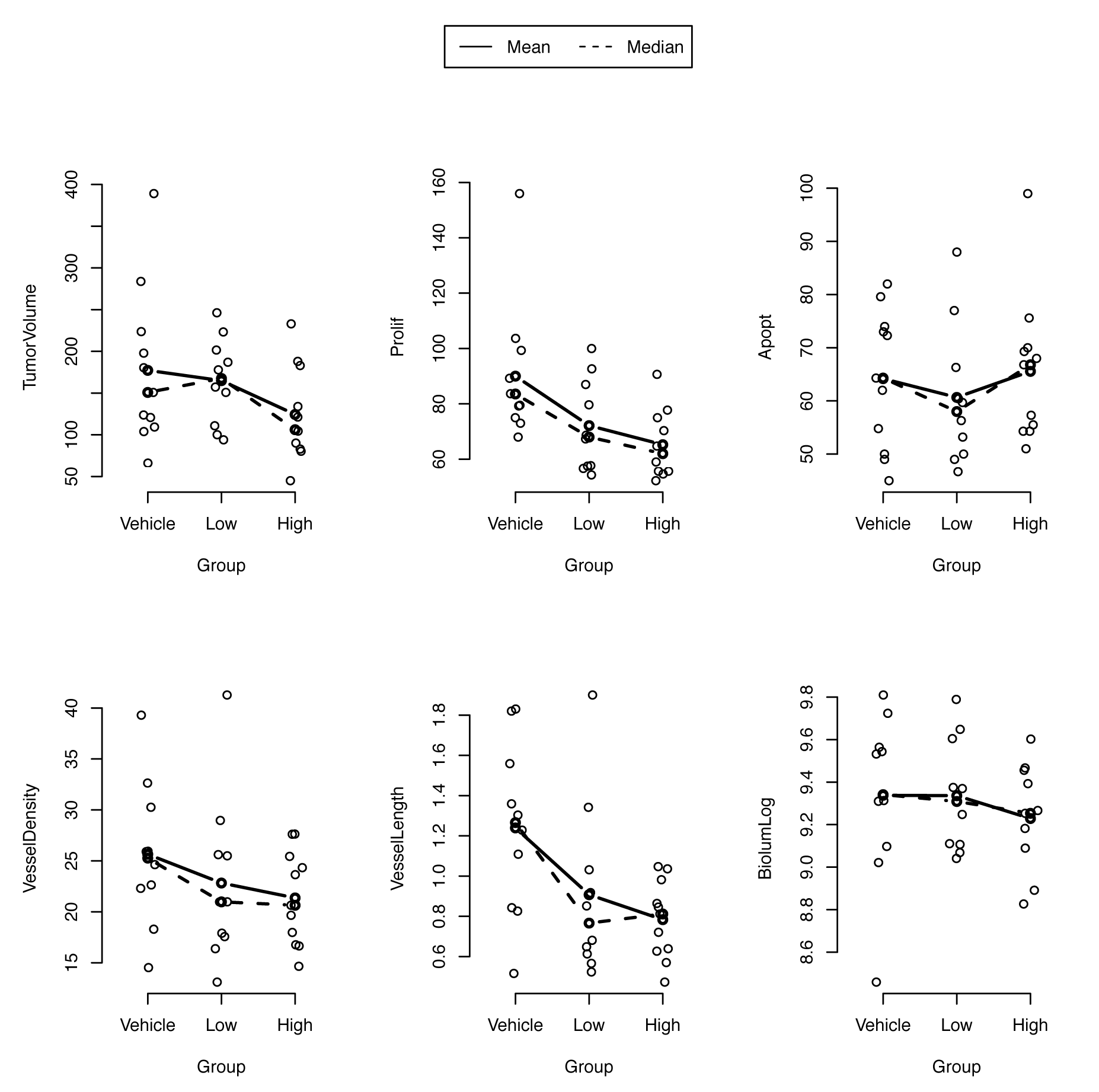

Supplement: Figure S2 — Univariate plots of the 6 end-point covariates. Means and medians are displayed in order to visualize the progressive trend for increasing dosage. (TIF) [file pone.0093764.s002.tif]

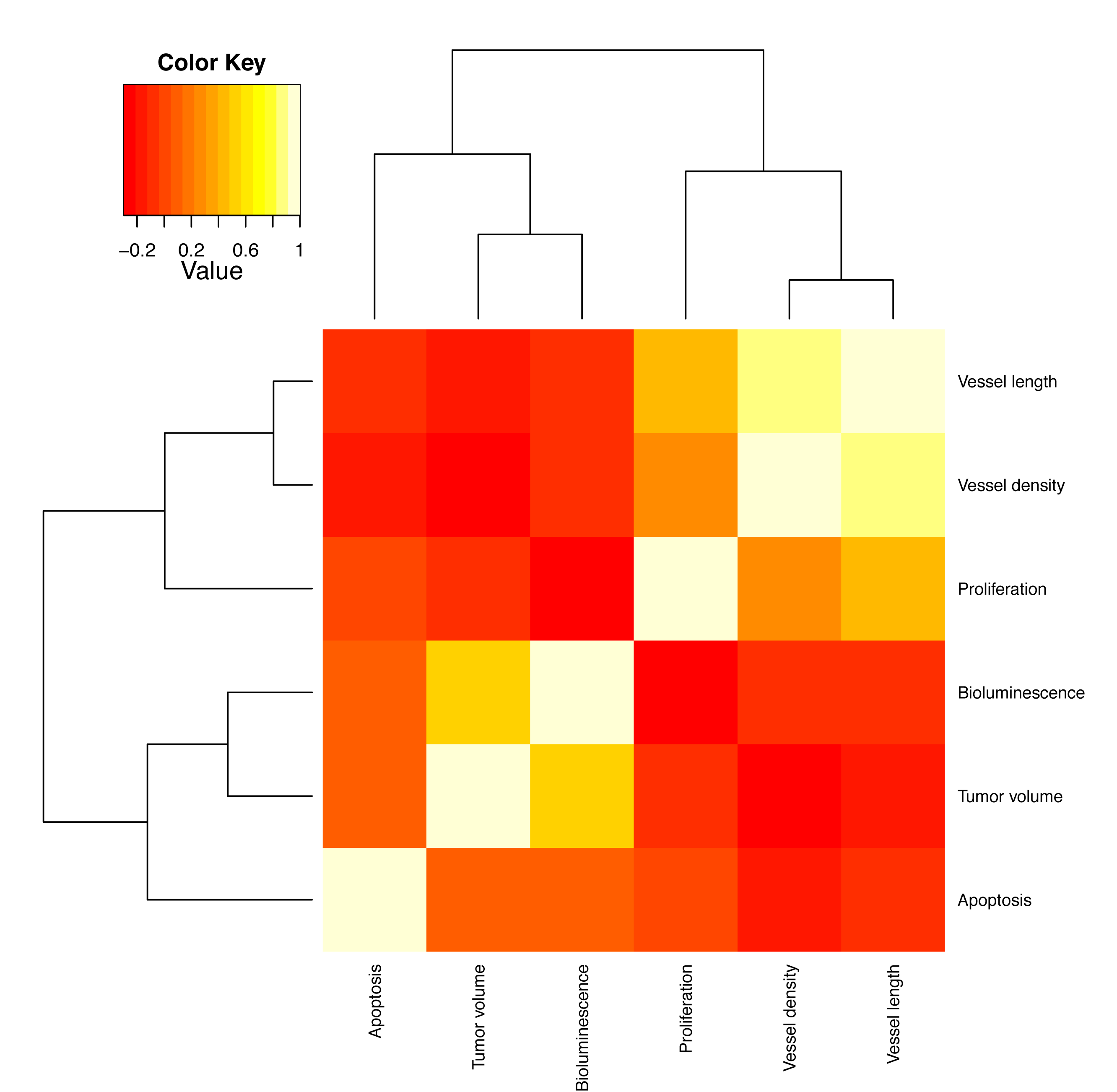

Supplement: Figure S3 — Covariance-variance matrix S scaled to a correlation matrix for visualization purposes. Agglomerative clustering of the factors illustrates that tumor volume and bioluminescence or vessel length and vessel density were correlated. (TIF) [file pone.0093764.s003.tif]
